# Beyond the plot: technology extrapolation domains for scaling out agronomic science

**DOI:** 10.1088/1748-9326/aac092

**Published:** 2018-05-14

**Authors:** Juan I Rattalino Edreira, Kenneth G Cassman, Zvi Hochman, Martin K van Ittersum, Lenny van Bussel, Lieven Claessens, Patricio Grassini

**Affiliations:** 1 Department of Agronomy and Horticulture, University of Nebraska-Lincoln, Lincoln, NE 68583-0915, United States of America; 2 CSIRO Agriculture and Food, 306 Carmody Road, St Lucia, QLD, 4067, Australia; 3 Plant Production Systems Group, Wageningen University, PO Box 430, 6700 AK, Wageningen, The Netherlands; 4 Soil Geography and Landscape group, Wageningen University and Research, PO Bo 47, 6700AA, Wageningen, The Netherlands; 5 International Institute of Tropical Agriculture (IITA), PO Box 10, Duluti, Arusha, Tanzania; 6 Author to whom any correspondence should be addressed

**Keywords:** geospatial analysis, technology extrapolation, research prioritization, impact assessment

## Abstract

Ensuring an adequate food supply in systems that protect environmental quality and conserve natural resources requires productive and resource-efficient cropping systems on existing farmland. Meeting this challenge will be difficult without a robust spatial framework that facilitates rapid evaluation and scaling-out of currently available and emerging technologies. Here we develop a global spatial framework to delineate ‘technology extrapolation domains’ based on key climate and soil factors that govern crop yields and yield stability in rainfed crop production. The proposed framework adequately represents the spatial pattern of crop yields and stability when evaluated over the data-rich US Corn Belt. It also facilitates evaluation of cropping system performance across continents, which can improve efficiency of agricultural research that seeks to intensify production on existing farmland. Populating this biophysical spatial framework with appropriate socio-economic attributes provides the potential to amplify the return on investments in agricultural research and development by improving the effectiveness of research prioritization and impact assessment.

## Introduction

Agronomy is the science of crop and soil management to produce food, fiber, and forage in a sustainable manner that does not deplete or degrade resources upon which future production depends. It is an applied ecological discipline that relies heavily on field experiments to identify improved farming methods involving interactions amongst crop rotation, crop variety, tillage practices, nutrient, water, weed, pest, and disease management, and their longer-term effects on soil properties that influence crop production. Each year billions of dollars are invested globally by the public and private sectors on agricultural research and development (Pardey *et al* 2016). Field experiments across many thousands of sites evaluating crop response to new technologies^7^ that result from this investment seek to identify those practices that raise yields, reduce risk, increase profits, and are more environmentally friendly. However, extrapolation of findings from these experiments to facilitate adoption by farmers is limited by the lack of a robust spatial framework to identify cropland ‘cohorts’ with similar soils and climate where a comparable response to a given set of technologies would be expected. Likewise, the ability to utilize these results to support more effective research prioritization and impact assessment is limited by the lack of an appropriate spatial upscaling method to estimate outcomes of technology adoption on crop production and natural resources at regional, national, and global scales (Grassini *et al* 2017, Kouadio and Newlands 2015). Hence, field-based agronomic research currently relies far too much on ‘trial and error’, which slows progress towards improved farm yields, profit, and environmental outcomes while also constraining effectiveness of research prioritization.

A robust spatial analysis framework that can delineate regions in which crop production technologies perform similarly would help address current limitations on extrapolation of results from agronomic field experiments. In principle, the impact of a particular agronomic technology, and the probability of adoption by farmers, should be predictable and of reasonably similar magnitude within a spatially defined region with similar biophysical (primarily weather and soil properties) and socio-economic (e.g. output and input prices, farm size, access to markets, credit, legislation and information) attributes. A unique combination of biophysical and socio-economic circumstances is hereafter referred to as a ‘technology extrapolation domain’ (TED). As a first step towards identifying a suitable TED framework, we focus on the biophysical attributes that define a TED for rainfed crop production while acknowledging the need to supplement this biophysical framework with appropriate socio-economic attributes.

While conceptually robust, the development of an appropriate biophysical TED framework has been an elusive goal for three reasons. The first concerns availability of good quality data of sufficient spatial coverage and resolution for climate and soil factors that have greatest influence on crop yields and response to management. These primary factors include rainfall and temperature regimes, as well as plant-available water holding capacity in the root zone (PAWHC^8^). Recent advances in database management and public accessibility of climate and soil databases with complete terrestrial coverage now make it possible to overcome this deficiency (Leenaars *et al* 2018, Soil Survey Staff 2016, van Wart *et al* 2013). A second and more difficult challenge reflects the need for a framework that strikes an effective balance between being too coarse such that variability of climate and soils within TEDs is so large that crop response to a given technology also varies, or too fine such that the benefits of aggregation are lost. Indeed, as noted by van Wart *et al* (2013), previous attempts to delineate TEDs have resulted in spatial frameworks that were too fine (Danvi *et al* 2016, Singh *et al* 1999) or too coarse (FAO 1978, Fischer *et al* 2002, Padbury *et al* 2002, Soil Survey Staff *et al* 2006, Wood and Pardey 1998) to be used to make agricultural research and development more efficient (see section S1 in supplementary material available at stacks.iop.org/ERL/13/054027/mmedia). Third, and perhaps most important, is the need to validate performance of a TED framework for ability to predict crop and cropping system performance. While several previous studies have evaluated the robustness of crop yield extrapolations and their uncertainty using both crop simulation and statistical models (e.g. Hochman *et al* 2016, Kouadio and Newlands 2015, van Bussel *et al* 2015), the focus of these previous efforts was on assessing the performance of these spatial frameworks at small geographic regions with approaches that require copious amount of data inputs. In contrast, the focus of this article is towards development and evaluation of a generic spatial framework that can be used to help prioritize agricultural research and development for sustainable intensification of crop production systems across spatial scales, from sub-national to national and global.

Although continuing trends of lower cost data storage and increased computing power, coupled with publicly available data bases on weather and soil properties with high spatial resolution may someday allow use of ‘customized’ extrapolation domains for evaluation of a specific technology or package of technologies, (e.g. fertilizer efficiency products, tillage practices, seeding rates, pest control measures, new crop cultivar or hybrid, new crops and crop rotations, and so forth), that capability is currently a bridge too far. Current knowledge and models are not sufficiently robust for development of such customized spatial frameworks, especially when multiple interacting technologies are involved. Until such customization is possible, the TED framework proposed here provides an initial, but substantive step towards the goal of greater efficiency and impact from investments in agricultural research worldwide, the more so because it is possible to make it more customized by adding other variables, including socio-economic variables, that are relevant for out-scaling and adoption of technology.

The objectives of this study were to: (i) develop extrapolation domains for technology transfer in cropping systems; (ii) perform a quantitative validation of the TED scheme for its ability to represent spatial variation in rainfed crop yield and the associated temporal variability; and (iii) demonstrate potential applications of the TED scheme. Although the TED framework can be applied worldwide, the evaluation of the TEDs requires detailed and spatially explicit data of weather, soil, crop management and yields. Hence, this paper reports initial testing of the framework in data-rich regions, including the US, Argentina, and Australia.

## Methods

We build on the spatial framework of the Global Yield Gap Atlas (www.yieldgap.org) (van Bussel *et al* 2015), which was developed to estimate crop yield gaps at local to global scales. A yield gap in rainfed agriculture is defined as the difference between: (i) potential rainfed yield when a crop is grown without limitations from nutrient deficiencies or pests and diseases, and (ii) actual yield obtained by the farmer. Potential rainfed yields are determined by rainfall, temperature regimes, and PAWHC, which also are dominant factors governing crop response to management technologies under rainfed conditions. Of these factors, the Global Yield Gap Atlas framework delineates climate zones (van Wart *et al* 2013) that account for rainfall and temperature regimes, but does not explicitly delineate PAWHC, which we include here as a primary categorical variable to define a TED. We hypothesize that the proposed framework is well suited for evaluation of crop response to new technologies because yields and yield stability (as quantified with the inter-annual coefficient of variation [CV]) in rainfed cropping systems are highly sensitive to these climate and soil factors (Lawes *et al* 2009, Williams *et al* 2016). Moreover, risk associated with temporal variation in climate is especially important in determining farmer adoption of new technologies (Koundouri *et al* 2006, Monjardino *et al* 2015).

The spatial framework we build utilizes four biophysical factors to delineate TEDs: (i) annual total growing degree-days, which gives an indication of the length of time during the year that crop growth is not limited by cold temperature; (ii) aridity index, which largely defines the degree of water limitation in rainfed cropping systems; (iii) annual temperature seasonality, which differentiates between temperate and tropical climates; (iv) PAWHC, which determines the capacity of a soil to store water to support crop growth during rain-free periods (see section S2 in supplementary material). At issue, then, is the degree of detail in PAWHC, as determined by the number of class intervals for this variable, to optimize spatial resolution for a robust TED framework. Hence, we develop TEDs at two levels of spatial resolution as determined by degree of detail in PAWHC categories (25- and 50 mm class intervals). The trade-off between degree of detail used to delineate TEDs and their applicability is illustrated by estimating the number of zones (TEDs) required to achieve a desired coverage of crop area.

We assess the performance of the spatial framework to define TEDs in terms of crop performance by evaluating average yields and temporal yield variability of maize across the US Corn Belt under the hypothesis that a robust spatial framework will adequately represent yield differences across a wide range of climate and soil types (see section S3 in supplementary material). The Corn Belt provides an appropriate region of focus to test this hypothesis because it represents 11% of continental US land area, accounts for 30 and 28% of global maize and soybean production (based on 2010– 2014 period) (FAOSTAT 2017, USDA-NASS 2016), and includes considerable variation in climate and soil properties that govern water holding capacity in the root zone (Grassini *et al* 2015). We evaluate the capacity of this spatial framework to account for variation in crop performance and management practices across spatial (a region with a wide range of climate and soil types) and temporal (years) dimensions using two different databases: (i) county-level maize yield data over a 10 year (2005–2014) time period (USDA-NASS 2016), and (ii) field-level soybean yield and management data from 3276 producer fields across the US Corn Belt collected over three years (2014–2016, see section S4 in supplementary material). We then demonstrate potential applications of the TED scheme in two ways (see section S5 in supplementary material). First, we use the spatial framework to demonstrate how to maximize coverage of crop production with a minimum number of testing locations for an existing field trial network. Second, we assess performance of an alternative cropping system that involves production of two crops per year in a TED in Australia, where most farmers currently only grow a single rainfed crop each year. This alternative cropping system with greater cropping intensity was identified in an analogue TED in Argentina where most farmers currently practice an annual rainfed double-crop system.

## Results and discussion

### Evaluating the proposed technology extrapolation domain framework

**Figure 1:**
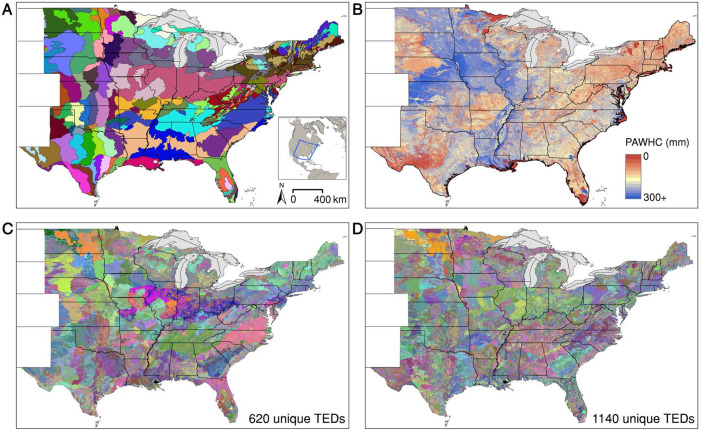
Climate zones, plant available water holding capacity and resulting technology extrapolation domains (TEDs). Maps of central-eastern US showing (*a*) climate zones, (*b*) plant available soil water holding capacity in the root zone, and (*c*) moderate and (*d*) high resolution TED schemes defined using different class intervals for plant-available water holding capacity in the root zone (50 and 25mm, respectively).

Estimating the number of zones required to achieve a desired coverage of crop area is essential for efficient evaluation of a new technology to ensure that field experiments are located in the most important production environments, as determined by climate and soil type. The challenge is to achieve maximum coverage of total crop production area with a minimum number of locations, which reduces costs and increases the extrapolation potential from investment in field research. For example, comparing two TED schemes that differ in degree of detail in class intervals used for PAWHC (figures 1(*c*) and (*d*), either 50 mm for a ‘moderate resolution’ scheme or 25 mm for a ‘high resolution’ scheme, gives a total of 620 (moderate resolution) and 1140 (high resolution) TEDs with in the central-eastern US, respectively. As a point of reference, an increase in crop water supply of 25 mm in PAWHC can support a cereal yield increase of about 0.5 Mgha^−1^ in regions like the Corn Belt where pre-plant rainfall is sufficient to fully recharge soil water holding capacity in most years but rainfall during the growing season does not meet crop water requirements (Grassini *et al* 2015). A 0.5 Mgha^−1^ yield increase is equivalent to about 5% of current average Corn Belt maize yields.

**Figure 2:**
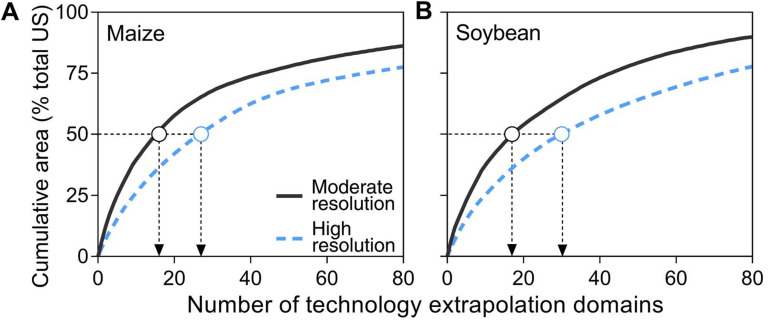
Crop area coverage as a function of number of moderate and high resolution technology extrapolation domains (TEDs). TEDs were sorted fromlargest to smallest according to their 2015 harvestedmaize (a) andsoybean(b) area. Black dashed lines indicate 50% of US national maize or soybean area coverage and downward arrows indicate the number of TEDs needed to achieve such coverage with each TED scheme. Total maize and soybean area in 2015 was 34 Mha and 33 Mha, respectively (USDA-NASS, 2017).

Whilst the finer class intervals would be more effective at detecting variation in yield response to management practices across different soil types, a trade-off emerges in the number of field studies located in unique TEDs required to achieve a desired coverage of crop area. Using rainfed maize and soybean as examples, the high resolution TED scheme requires field studies in 27 and 30 unique TEDs for US maize and soybean, respectively, to reach 50% coverage of total production area for both crops, *versus* field experiments in only 16 and 18 unique TEDs for the moderate resolution scheme (figure 2). Achieving a level of coverage above 50% of total maize or soybean area requires an increasingly greater number of field studies in additional TEDs because the relative contribution of each additional TED follows a strong diminishing return, especially with the high resolution scheme.

At issue, then, is the degree of spatial resolution needed to adequately represent crop and cropping system performance for technology evaluation. Lack of spatially congruent datasets for crop performance, however, makes it difficult to compare TED schemes with different spatial resolution. For example, average TED size in the high resolution scheme is 4000 km^2^, and, in many cases, the TEDs are smaller than counties, which is the smallest spatial scale at which US maize yields are reported. In contrast, average TED size in the moderate resolution scheme is 7600 km^2^, which is roughly the same size as large counties. Therefore, we used the moderate resolution scheme portrayed in figure 1(*c*) to evaluate maize yield and temporal yield variability, quantified by the CV in yield across climate zones and soil types in the US Corn Belt based on annual county-level data from 2005–2014 (USDANASS 2016). For this analysis, counties were grouped within the same TED if *>*50% of the maize area in those counties was located within the same unique TED. Because most US maize farmers use fertilizers and modern pest control measures to minimize yield losses from nutrient deficiencies and pest damage, average rainfed yields are relatively high at about 70% of potential rainfed yields (van Wart *et al* 2013), which means that weather and PAWHC, as governed by the categorical variables delineating TEDs rather than socio-economic variables, have a dominant influence on yields. The CV provides a measure of risk due to impact of year-to-year variation in water supply as determined by weather and PAWHC, and can be used as an indicator for yield stability.

**Figure 3:**
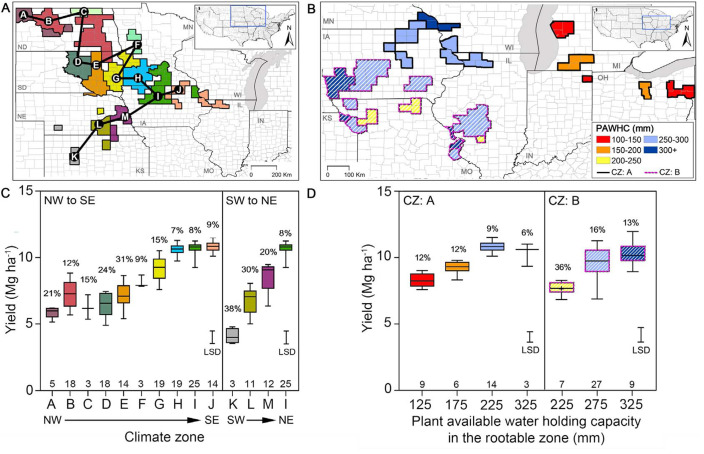
Average maize yield and its temporal variability across climate zones and soil types in the US Corn Belt. Map of the central-eastern US showing groups of counties with (*a*) similar plant-available water holding capacity in the root zone (PAWHC) but located in different climate zones (each color corresponds to a different climate zone) and (*b*) similar climate zones ((*a*) and (*b*): solid and hatched colors within counties, respectively) but different PAWHC (each color corresponds to a different PAWHC). Connecting lines in (*a*) correspond to NW-SE and SW-NE transects. Letters in (*c*) were assigned to climate zones following the NWto SE or the SWto NE directions. Variation in average maize yields, within and amongst the groups of counties defined in (*a*) and (*b*), is shown in (*c*) and (*d*), respectively, using box plots. Box indicates 25th, 50th, and 75th percentiles; error bars indicate minimum and maximum yield within a given class. Values above horizontal axis in (*c*) and (*d*) indicate number of counties within each climate zone or PAWHC class, respectively. Percentage values above box plots indicate the inter-annual coefficient of variation. LSD bar represents the least significant difference (*p* = 0.01) among average yields for each climate zone (*c*) or PAWHC(*d*).

Average yield and yield stability were evaluated across groups of selected counties in two dimensions, temporal and spatial, to assess the capacity of the TED framework to account for variation in crop performance across biophysical environments and years. The spatial analysis was performed along two transects (i) with different climate but with soil in the same PAWHC class (figures 3(*a*) and (*c*), and (ii) with soils with different PAWHC class within the same climate zone (figures 3(*b*) and (*d*). Results show that differences in average yields and associated CVs vary in a manner consistent with expectations due to climate and soil type. For example, counties with similar PAWHC (250–300 mm) but located in different climate zones exhibited significant differences in average yield and CV across two directional transects in the US north-central region (figure 3(*c*)). In the NW to SE direction, both average yield and yield stability increase towards the SE due to longer growing season and smaller aridity index (i.e. greater water supply). In the SW to NE direction, yields and yield stability likewise increase due to smaller aridity index. Similarly, counties located in the same climate zone, but with different PAWHC exhibited increasing yields and decreasing CVs in TEDs with greater PAWHC (figure 3(*d*)). These trends are consistent with trends in rainfed yield potential as simulated with a well-validated maize simulation model that accounts for the effects of rainfall, temperature regime, and PAWHC (Grassini *et al* 2009). In addition, a stepwise multiple regression analysis identified three of the four categorical factors that delineate TEDs as significant variables that together explain 56% and 37% of the observed variation in county average farm yield and CV, respectively (supplementary table S1).

Likewise, temporal analysis of county-level maize yield indicated that the TED framework accounts for differences in yield across the majority of years evaluated (see section S4 in supplementary material). This finding agrees with results from the analysis of field level soybean yield and management practices across the Corn Belt (cultivar, tillage and pesticide use), which shows that 80%–99% of the variance (excluding the error term) in yield and management can be explained by the TEDs alone, while the year term (including TED x year) explained *<*20%of the variation in all cases (see section S4 in supplementary material). To summarize, the TED framework was satisfactorily evaluated on its ability to distinguish regions with different yield level, yield stability, and management practices for two crops (maize and soybean), across two spatial scales (county and field-level), and two dimensions (temporal and spatial) over a large geographic region with diversity in climate and soil that account for about one-third of global maize and soybean production. We are not aware of previous efforts to quantitatively evaluate the effectiveness of a spatial framework for characterizing performance of crop production in this manner across both time and space.

These results suggest that the moderate resolution TED scheme is robust for capturing the influence of key biophysical factors on crop productivity and its variability and, by extension, to also capture differences in crop response to crop and soil management practices that depend on the amount and reliability of water supply and length of growing season in rainfed cropping systems. By contrast, a random selection of counties did not identify differences in yield and CV among regions(supplementaryfiguresS1 andS2).And while a higher resolution TED scheme would increase precision in discerning the directional trends shown in figures 3(*c*) and 3(*d*), the greater precision comes at the expense of much larger costs associated with the increasing number of field studies required to evaluate new technologies at different locations to achieve a desired level of coverage in total crop area.

### Technology extrapolation domains as a tool to guide evaluation and scaling out

**Figure 4:**
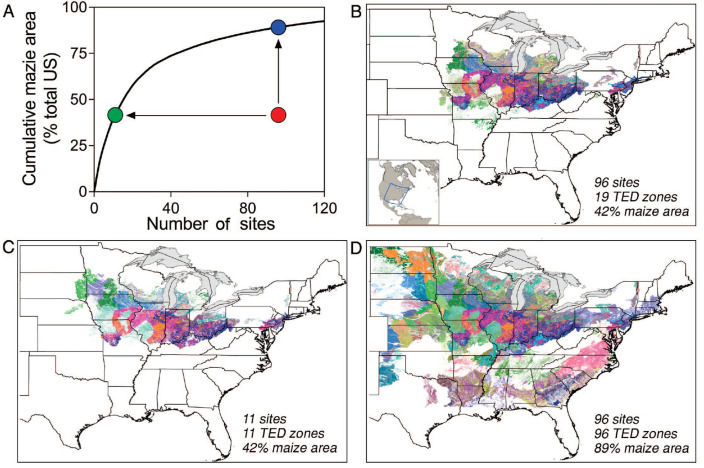
Strategic choice of number and location of field experiments. Strategic location of field experiments to maximize coverage of unique technology extrapolation domains (TEDs) based on a network of 96 field experiments conducted in 2015 and located in farmers’ fields to evaluate a product that improves fertilizer use efficiency of rainfed maize. (*a*) The efficiency frontier shown by the curvilinear line representing maximum maize crop area coverage for a given number of sites if each experiment was allocated in a unique TED unit, starting fromthe TED with largest crop area on the left and sequentially smaller crop area to the right. Mapping the 96 sites showed that many were located in the same TED such that only 19 unique TEDs and 42% of total maize area was covered as indicated by the red dot in (*a*). TEDs in which field trials were located are shown in (*b*). Strategic placement of field experiments such that each was located in a unique TED reduces the number of trials by 90% to give the same crop area coverage within unique TEDs as shown in (*c*) and the green dot in (*a*), while the same number of field experiments would more than double the crop area coverage as shown in (*d*) and the blue dot in panel (*a*).

Given the high cost of time and labor to implement replicated field studies in commercial production fields, the TED framework presented here can help (i) optimize the number of environments covered by a field trial to maximize the crop area coverage in unique TEDs for a given number of sites or, alternatively, to reduce the number of sites without sacrificing crop area coverage in unique TEDs, (ii) select specific environments for testing a technology where it is most likely to have the greatest impact based on biophysical attributes of the selected TEDs, (iii) delineate the extrapolation domain for specific field trials, allowing up-scaling of expected impact from trial locations to TEDs in which the trials were conducted, and (iv) facilitate technology transfer across analog TEDs located in different geographic regions. Potential to improve efficiency of a field experimentation program is illustrated in figure 4(*a*). The curvilinear line represents the crop production area coverage for a given number of field trials if each site is located in a unique TED, starting from the origin with TEDs that include largest crop production area to those with smallest area to the right. Hence, any set of field experiments can be compared against this ‘efficiency frontier’ line to identify opportunities for greater coverage of crop area within unique TEDs.

To illustrate this point, we evaluate maize area coverage by a set of 96 field experiments conducted in 2015 and established in farmer fields to evaluate a product thought to improve nitrogen fertilizer efficiency of maize^9^ (supplementary figure 4). The 96 sites were located within 19 TEDs (figure 4(*b*)) which accounted for 42% of total US maize area (figure 4(*a*)). In contrast, strategic reallocation of each field experiment in a unique TED with greatest crop area would achieve the same coverage with only 11 field studies (figure 4(*c*)), or double the coverage if each of the 96 trials would be reallocated in a unique TED (figure 4(*d*)).

This case study relies on a number of assumptions. It assumes that one site per TED is enough to capture crop response to a given technology within that TED. It also assumes no risk of losing sites to unforeseen events such as flooding, hail, or heavy yield loss from factors such as diseases, insect or pests. It may be worthwhile to have more than one site per TED to account for unforeseen events and to have a strategic focus on TEDs with largest crop area or where the technology being tested is expected to have greatest potential impact on yields, profit, and environmental quality. In addition, this TED framework for site selection can easily be expanded to include other variables that have influence on performance of a given technology or its adoption by farmers, including irrigation, other biophysical factors such as soil pH, organic matter content, or terrain slope as well as socio-economic factors such as distance to market, farm size, and so forth.

### Extrapolating technology across large distances

**Figure 5:**
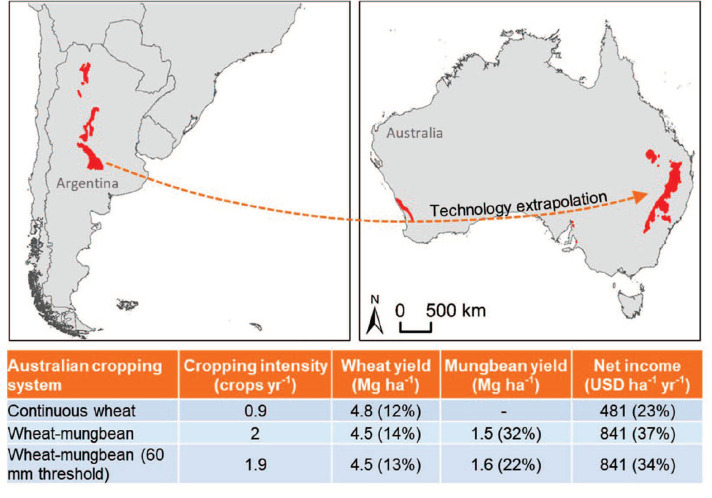
Technology evaluation in the same technology extrapolation domains (TED) on different continents. Argentinean and Australian maps showing a unique climate zone (in red) shared by both countries. Within the climate zone in each country, zones with similar plant-available water holding capacity in the root zone were compared (i.e. the same TED). Table shows the performance of three alternative cropping systems of varying intensity in Gunnedah (Australian location within the TED) in term of simulated water-limited yield potential, yield variation (CV, in brackets), annual net income (expressed as gross income minus variable and overhead costs) and variation in annual income (CV, in brackets).

Another application of the described spatial framework is to compare cropping systems in the same TED across different regions, countries and continents that share a unique TED. For example, it is possible to evaluate promising technologies to improve resource capture and productivity of land and water resources that have been widely adopted by farmers in one region but have not yet been tested or are not widely used, in an analog TED elsewhere. This hypothesis was explored for a TED that is present in both Argentina and Australia (figure 5). In Australia, a cropping intensity of 0.9 rainfed crop per year (this crop may be a winter crop such as wheat, barley or chickpea or a summer crop such as sorghum, maize or mungbean) is currently the dominant cropping system (Hochman *et al* 2014). Double cropping is a rare and opportunistic practice in this TED. In contrast, 1.5 crops per year are grown in the analogue TED in Argentina where a two-yr rotation of soybean-wheat-soybean-fallow is common, resulting in greater efficiency in utilization of water and solar radiation, and larger total yield when expressed on an annual basis (supplementary table S4).

Given this large difference in cropping systems within the same TED on different continents, we investigated the feasibility of increasing annual productivity and resource capture in the Australian TED by increasing crop intensity through inclusion of a summer legume (mungbean) in the traditional single crop per season (here represented as wheat-fallow system). Compared to soybean, mungbean is more suited to this Australian TED because its growth period, from sowing to harvestable maturity, better fits the period of time when there is sufficient stored soil moisture and rainfall to support crop growth. Simulation analysis revealed that an annual rainfed double-crop of wheatmungbean (i.e. 2 crops per year) would be a superior alternative to the traditional crop-fallow system that currently dominates in the analogue Australian TED. While the wheat-fallow system exhibited highest yield for a single wheat crop, it had much lower annual net income relative to the wheat-mungbean rotation (figure 5). And although mungbean yields in the rotation were less stable than for a single crop of wheat (CV= 32% versus 12%), the higher risk can be reduced by sowing mungbean only when soil water status at sowing is above a minimum threshold of ≥ 60 mm available soil water in the root zone. Hence, the intensified wheat-mungbean cropping system improves resource capture and increases Australian producer income by 75% relative to the current wheat-fallow system although year-to-year variability in net income is greater. Remarkably, a relatively small number of farmers in the Australian TED have recently started to include opportunistic cropping of mungbean within the traditional wheat monocrop system (Rachaputi *et al* 2015), which adds confidence to broad applicability of this type of cropping system analysis across analogue TED zones as a tool for technology evaluation and transfer.

## Conclusions

Combining high yields with efficient use of resources and small environmental footprint represents a major challenge and will require adequate investments in research and development, as well as efficient and effective prioritization of these investments. Likewise, once new technologies are developed, new approaches are needed to drive more rapid technology transfer to achieve widespread adoption in other regions. A robust spatial framework for evaluating the ‘extrapolation domain’ for new technologies represents an essential tool for achieving these goals. Such a framework for technology testing and transfer must account for the most important factors governing crop productivity and environmental performance without requiring an excessive number of variables and categories. To that end, results from evaluation of the TED framework presented here show promise for capturing effects of dominant climate and soil factors responsible for variation in rainfed crop yields, and for facilitating greater efficiency in testing of new technologies and gaining adoption of those that improve yields, yield stability, and profits while reducing negative environmental impacts through delineating the area where they are likely to work best. An online version of the TED framework that focuses on US maize production, including tools to select TEDs based upon different attributes (geographic location, biophysical factors, crop harvested area), is available at http://nutrientstar.org/about-teds/. The shapefile delineating the TED spatial framework is available for downloading at: www.yieldgap.org/cz-ted.

## Footnotes

7 Here we use technology in a broad sense to include single factors such as sowing dates, tillage methods, crop varieties, fertilizer, irrigation, and pest management practices, as well as system-level innovations such as a new crop rotation, an alternative crop, and inclusion of cover crops.

8 PAWHC (in millimeters) represents the capacity of soil to store water to support crop growth. The size of this water reservoir determines the degree to which a soil can buffer against transient periods of water deficits when rainfall does not meet crop water requirements. PAWHC depends on the depth to which roots can grow, as determined by soil physical and chemical properties, and on soil particle size (called soil texture). While other soil factors are also important, such as nutrient stocks, pH, salinity, and soil porosity, these can be modified by management and are therefore considered secondary factors and are not used to delineate TED boundaries.

9 Field experiments were implemented by the NutrientStar. evaluation program (www.nutrientstar.org).

## References

[cit0001] Danvi A et al 2016 A spatially explicit approach to assess the suitability for rice cultivation in an inland valley in central Benin *Agric. Water Manage*. 177 95–106

[cit0002] FAO 1978 Report on the agro-ecological zones project *World Soil Resources Report 48* (Rome)

[cit0003] FAOSTAT (2017). Crops and livestock trade database.

[cit0004] Fischer G et al 2002 *Global Agro-Ecological Assessment for Agriculture in the 21st Century: Methodology and Results* (Laxenburg: IIASA)

[cit0005] Grassini P et al 2017 Robust spatial frameworks for leveraging research on sustainable crop intensification *Glob. Food Secur*. 14 18–22

[cit0006] Grassini P et al 2015 High-yield maize–soybean cropping systems in the US Corn Belt *Crop Physiology. Applications for Genetic Improvement and Agronomy* ed Sadras V O and Calderini D F (Oxford: Academic) pp 15–42

[cit0007] Grassini P et al 2009 Limits to maize productivity in Western Corn-Belt: a simulation analysis for fully irrigated and rainfed conditions *Agric. Forest Meteorol*. 149 1254–2165

[cit0008] Hijmans R J et al 2005 Very high resolution interpolated climate surfaces for global land areas *Int. J. Clim*. 25 1965–78

[cit0009] Hochman Z et al 2016 Data rich yield gap analysis of wheat in Australia *Field Crops Res*. 197 97–106

[cit0010] Hochman Z et al 2014 Crop sequences in Australia’s northern grain zone are less agronomically efficient than implied by the sum of their parts *Agric. Syst*. 129 124–32

[cit0011] Kouadio L and Newlands N K 2015 Building capacity for assessing spatial-based sustainability metrics in agriculture *Decis. Anal*. 2 2

[cit0012] Koundouri P et al 2006 Technology adoption under production uncertainty: theory and application to irrigation technology *Am. J. Agric. Econ*. 88 657–70

[cit0013] Lawes R A et al 2009 Integrating the effects of climate and plant available soil water holding capacity on wheat yield *Field Crops Res*. 113 297–305

[cit0014] Leenaars J G B et al 2018 Mapping rootable depth and root zone plant-available water holding capacity of the soil of sub-Saharan Africa *Geoderma* 324 18–36 30122789 10.1016/j.geoderma.2018.02.046 PMC5913732

[cit0015] Monjardino M et al 2015 Farmer risk-aversion limits closure of yield and profit gaps: a study of nitrogen management in the southern Australian wheatbelt *Agric. Syst*. 137 108–18

[cit0016] Padbury G et al 2002 Agroecosystems and land resources of the Northern Great Plains *Agron. J.* 94 251–61

[cit0017] Pardey P G et al 2016 Agricultural R&D is on the move *Nat. Comment* 537 301–3 10.1038/537301a 27629624

[cit0018] Rachaputi R C N et al 2015 Physiological basis of yield variation in response to row spacing and plant density of mungbean grown in subtropical environments *Field Crops Res.* 183 14–22

[cit0019] Singh V P et al 1999 Ecosystem analysis-based methodology for technology extrapolation *Resource Management in Rice Systems: Nutrients* ed Balasubramanian V et al (Dordrecht: Springer) pp 213–29

[cit0020] Soil Survey Staff 2016 *National Value Added Look Up (valu) table Database for the Gridded Soil Survey Geographic (gSSURGO) Database for the United States of America and the Territories, Commonwealths, and Island Nations served by the USDA-NRCS* (United States Department of Agriculture, Natural Resources Conservation Service)

[cit0021] Soil Survey Staff 2006 Land Resource Regions and Major Land Resource Areas of the United States, the Caribbean, and the Pacific Basin. Agricultural Handbook 296 digital maps and attributes

[cit0022] USDA-NASS 2016 USDA-National Agricultural Statistics Service (NASS), Crop US State and County Databases (www.nass.usda.gov)

[cit0023] USDA-NASS 2017 USDA-National Agricultural Statistics Service (NASS), National Cultivated Layer (www.nass.usda.gov)

[cit0024] van Bussel L G J et al 2015 From field to atlas: upscaling of location-specific yield gap estimates *Field Crops Res*. 177 98–108

[cit0025] van Wart J et al 2013 Use of agro-climatic zones to upscale simulated crop yield potential *Field Crops Res*. 143 44–55

[cit0026] Williams A (2016). Soil water holding capacity mitigates downside risk and
volatility in US rainfed maize: Time to invest in soil organic
matter?. *PLoS ONE*.

[cit0027] Wood S and Pardey P G 1998 Agroecological aspects of evaluating agricultural R&D *Agric. Syst*. 57 13–41

