# Supplementary material for: Beyond the plot: technology extrapolation domains for scaling out agronomic science
Source: Environ Res Lett. 2018 May 14;13(5):054027. doi: 10.1088/1748-9326/aac092 (PMC7691761; doi:10.1088/1748-9326/aac092)
Supplement: Supplementary file 1 [file ERL-13-05-054027-s001.pdf]

SUPPLEMENTARY MATERIALS

S1. Previous studies on extrapolation domain for agriculture

Most previous studies aiming to develop extrapolation domains in agriculture can be roughly grouped into two categories. One category includes studies on extrapolation domains developed for specific regions or crops, typically based on a large number of biophysical attributes or field-level yield data that are most relevant to the specific crop-country case, but not representative at larger spatial scales or not sufficiently flexible to be applied more broadly across different crops, cropping systems, and regions with different climate and soil properties (Danvi et al., 2016; Kouadio and Newlands, 2015; Singh et al., 1999). Another category of studies focuses on extrapolation domains that are global but based on very coarse-scale climatic and soil properties that lack sufficient spatial resolution to be of agronomic relevance (FAO, 1978; Fischer et al., 2002; Padbury et al., 2002; Wood and Pardey, 1998).

S2. Development of technology extrapolation domain framework

The TED framework was created by combining the GYGA[1] climate zone scheme (van Wart et al., 2013) with spatial distribution of plant-available water holding capacity in the root zone (PAWHC, Figure 1B) (Soil Survey Staff, 2016). The latter is a measure of how much water the soil can store and make available to plants during rain-free periods. For example, a typical US Corn Belt soil can store 250 mm of plant-available water in the maize root-zone, which is enough to support crop growth without severe water stress during rain-free periods for about 3 weeks during the period of peak water demand in July and August. Other soil properties such as

[1] Global yield gap atlas (www.yieldgap.org)

pH and nutrient stocks can also affect crop performance and technology transfer but these traits can be modified by management and therefore were not included as a categorical variable in the TED scheme.

The GYGA climate zone scheme covers the entire terrestrial surface (van Wart et al., 2013). Each climate zone is a combination of (i) annual total growing degree-days, (ii) aridity index, and (iii) temperature seasonality. Annual total growing degree-days were calculated following Licker et al. (2010) as the cumulative annual average temperature with a non-crop-specific base temperature (0 ˚C). Aridity index was estimated as the annual total precipitation divided by annual total potential evapotranspiration. Temperature seasonality was calculated as the standard deviation of monthly average temperatures. Both growing degree-days and temperature seasonality were calculated using climate data from WorldClim (Hijmans et al., 2005) and the aridity index was taken from GIAR-CSI (Zomer et al., 2008). Although the GYGA climate zones do not account for precipitation seasonality, it has the smallest within-domain variability for this variable compared with other agro-climatic schemes (van Wart et al., 2013). Ranges of each of these variables, excluding those terrestrial areas where cropland area occupies <0.5% of total area, were divided into 10 interval classes for growing degree days and aridity index and 3 interval classes for temperature seasonality which, when combined in a grid matrix, resulted on 300 possible climate zones. Relative to other agro-climatic schemes, the GYGA climate zone scheme achieved the best compromise between number of climate zones and low climate variability within each climate zone (van Bussel et al., 2015; van Wart et al., 2013).

In the present study, very small isolated portions of climate zones (‘inclusions’), likely a consequence of artifacts in the underpinning data or methods to build the climate zones, were removed from the original central-eastern US climate zone scheme. Criteria to remove these inclusions were (i) area < 350,000 ha, (ii) surrounding climate zone, at least, five times larger than inclusion area, and (iii) standard deviation for terrain elevation <10% (USDA-FSA-APFO, 2016). These rules aimed to discard inclusions attributable to an artefact of climate zones computations while keeping real microclimates generated by sudden changes in temperature and precipitation due to terrain elevation or water bodies. A total of 416 inclusions were removed, including 10 small climate zones. The ‘refined’ climate zone scheme for central-eastern US had a total of 91 climate zones (Figure 1A).

Refined climate zones were combined with two maps of PAWHC to create TED scheme with moderate and high spatial resolution. The moderate resolution scheme (Figure 1C) was created by intersecting the refined climate zones map with a 50-mm PAWHC class interval map (total of 7 PAWHC classes). The high resolution scheme (Figure 1D) was created by intersecting the refined climate zones map with a 25-mm PAWHC class interval map (total of 13 PAWHC classes). Therefore, these two frameworks differed in the degree of detail to which soil types are classified according to their capacity to store water. Roughly, the two PAWHC class intervals (25 and 50 mm) are equivalent to 0.5 and 1 Mg ha^{-1} of cereal yield, respectively. Both TED schemes had a grid resolution of 250 x 250 m. Maize and soybean 2015-harvested area (USDA-NASS, 2017) located within each individual TED was determined separately for the high and moderate resolution schemes.

S3. TED framework validation

Actual rainfed maize yield (2005-2014) and its inter-annual variation at county-level were used to validate the TED framework (USDA-NASS, 2016). Inter-annual variation was quantified with the coefficient of variation (CV, expressed as percentage) as the quotient between standard deviation of the mean and average yield. Validation consisted in evaluating changes in maize actual yield and CV among groups of counties belonging to the same TED. Only counties with >50% of their maize area located within a unique TED were included in the analysis. Validation was performed for the moderate resolution TED framework, which has larger area per TED than the high resolution TED framework (7600 km^2 versus 4000 km^2 on average), in order to include more counties in the analysis (Figure 3). A total number of 239 US counties were selected, and aggregated by TED. Changes in yield and CV were evaluated across groups of counties with (i) different climate zones within the same soil type (Figure 3A, C), and (ii) different soil types within the same climate zone (Figure 3B, D). Average yield and yield variation were computed for each group of counties. Distributions of county average yield and CV, within the same climate zone or PAWHC class, were shown in box plots. Data were analyzed by analysis of variance and means were compared by least significant differences (LSD) test at the P=0.01 significance level. Additionally, as a control test, we created groups of randomly selected counties to evaluate what would have been the variation in yield and CV among counties without the aggregation based upon the TED framework. Results were also presented in box plots (Figure S1 and S2).

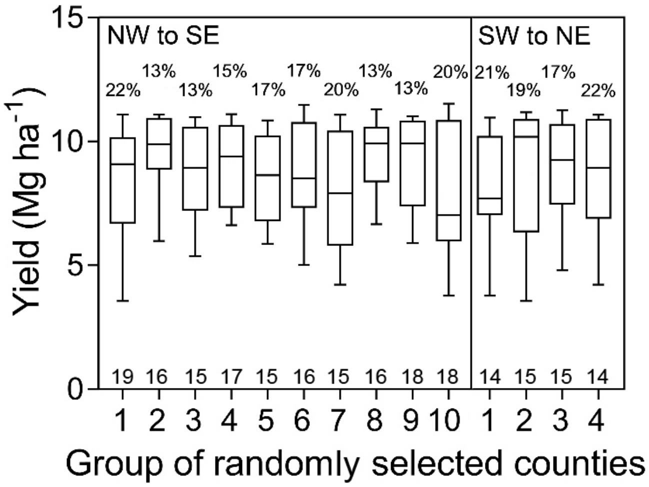

Figure S1. Yield variation among groups of randomly selected US counties. *Variation of maize yields in different groups of counties randomly selected from the pool of counties designated to validate the technology extrapolation domain in Figure 3A. Box indicates 25th, 50th, and 75th percentiles and error bars indicate minimum and maximum yield. Values below boxes indicate the number of counties in each group and percentage value above the box plots indicate the inter-annual coefficient of variation for yield. This figure illustrates that a random selection of counties did not identify differences in yield and CV among regions as compared with Figure 3A.*

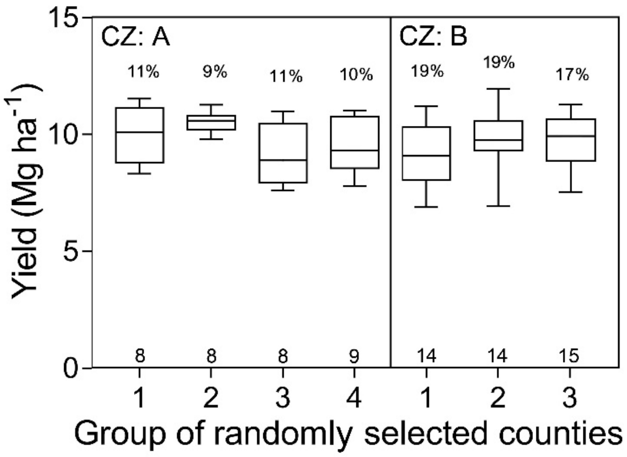

Figure S2. Yield variation among groups of randomly selected US counties. *Variation of maize yields in different groups of counties randomly selected from the pool of counties designated to validate the technology extrapolation domain in Figure 3B. Box indicates 25th, 50th, and 75th percentiles and error bars indicate minimum and maximum yield. Values below boxes indicate the number of counties in each group and percentage value above the box plots indicate the inter-annual coefficient of variation for yield. This figure illustrates that a random selection of counties did not identify differences in yield and CV among regions as compared with figure 3B.*

Stepwise multiple-regression analysis was performed to explain the variation in grain yield and yield CV (dependent variables) among counties that belong to different TEDs (independent variables) (Kleinbaum et al., 1998). The objective was to validate the TED scheme to determine whether using TED biophysical parameters (aridity index, annual total growing degree days, temperature seasonality, and PAWHC) can explain significant variations in yield and CV. All TED biophysical features were used for the analysis except for temperature seasonality, which was identical amongst the counties included in the TED validation. Stepwise regressions were performed using all counties and years included in the TED validation (n = 239). Parameters derived from the analysis included adjusted r^2, parameter estimates (and their standard errors), T-test and P values (Table S1). Yield and CV were transformed to reduce negative effect of non-normal distribution on ANOVA and regression analysis. However, results derived from transformed data did not change much from those resulting from the original ones, so that we publised non-transformed data.

Table S1- Stepwise regression parameters of the relationship between yield (actual yield and its variation) and technology extrapolation domain (TED) components. *Parameters are the estimate of each TED components, standard error, T value and P value. All variables used to develop the TEDs (GDD, aridity index, and PAWHC) where included into the analysis except for temperature seasonality which did not differ among the counties selected for the TED validation.*

Variable	Adjusted r^2	TED components	Estimate ± standard error	T value	P value
Yield (Mg ha^{-1})	0.56	Intercept	3.71 ± 0.91	-4.1	<0.0001
		PAWHC	0.015 ± 0.002	18.6	<0.0001
		GDD	-0.0007 ± 0.0001	6.9	<0.0001
		Aridity index	0.0011 ± 0.00006	-4.5	<0.0001
Yield CV (%)	0.37	Intercept	29.22 ± 6.25	4.7	<0.0001
		PAWHC	-0.044 ± 0.014	-3.1	0.0023
		GDD	0.011 ± 0.001	10.6	<0.0001
		Aridity index	-0.004 ± 0.0004	-10.0	<0.0001

TED: technology extrapolation domain; PAWHC: plant-available water holding capacity in the root zone (in millimeters); GDD: annual total growing degree days (in °Cd); CV: coefficient of variation

S4. Evaluation of the TED framework across years

We assessed the robustness of the spatial framework by analyzing the degree to which the relationship between TEDs and yield and management changes across years. The analysis was performed using two independent data sources: (i) county-level data on maize rainfed yield reported over a 10-year (2005-2014) time period, including a total of 1895 county-year cases (USDA-NASS, 2016; see section S3 in Supplementary Material) and (ii) field-level soybean data on yield and management practices (cultivar choice, tillage and pesticide use) from 5331 producer fields collected over three crop seasons (2014-2016) across the US North-Central region (Figure S3, A). Briefly, producers were asked to report the range of average field yields across the fields sown with soybean in each year and to provide data for a number of fields that portray well that yield range. Requested data included field location, average field yield (at 13% seed moisture content), crop management (*e.g.,* cultivar, tillage method, pesticides), and incidence of unforeseen events (*e.g.,* hail, waterlogging, and frost). Survey data were screened to remove erroneous, incomplete data entries, and fields severely affected by any of the aforementioned adversities. Surveyed fields were grouped based upon their climate and soil domain using the TED spatial framework presented here. We selected those TEDs with >100 fields, which resulted in 17 TEDs that portrayed the diversity of climate and soils in the US North Central region (Figure S3, B) and represented *ca.* 40% of total US soybean area. After quality control and TED selection, the database contained a total of 3276 fields sown with soybean during 2014-2016, with an average of 193 fields per TED.

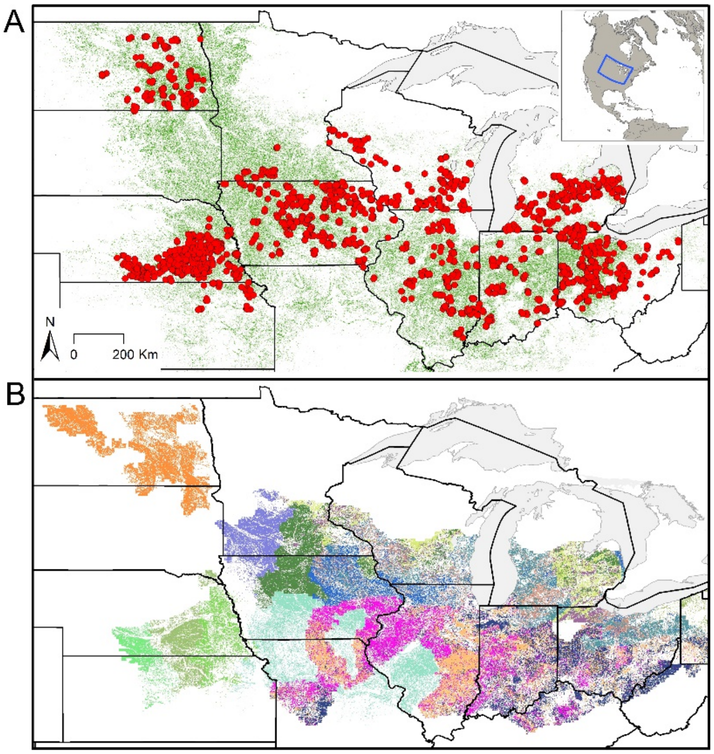

Figure S3. Field-level soybean data location and its extrapolation domains. *Map of the North-Central US region sowing the (A) soybean harvested area in year 2015 (green area; USDA-NASS) and location of the 3276 surveyed soybean fields (red dots) collected during 2014-2016, and (B) 17 technology extrapolation domains (TEDs) with >100 fields per TED (193 fields per TED on average). Toghther, the 17 selected TEDs covered 13.3 Mha of soybean and represented ca. 40% of total US soybean area.*

Analysis of variance (ANOVA) of yields and management practices was conducted to examine the partitioning of sum of squares amongst year, TED, and TED × year sources of variation. ANOVA residuals were taken as a measure of the county-to-county (county-level maize database) or field-to-field (soybean field-level database) variability within TED. Analysis of the USDA-NASS data showed that TEDs accounted for 81% of the treatment sum of squares (excluding the error), and explained four times more than the contribution of year and TED x year (Table S2).

Table S2 – Analysis of variance of the TED framework across years using county-level maize yield data. *Analysis of variance for actual rainfed maize yield at county level during 2005–2014 years across 20 technology extrapolation domains (TEDs) located in the US Corn Belt region. A total number of 239 US counties were selected, and aggregated by TED. Only counties with >50% of their maize area located within a unique TED were included in the analysis.*

Variable (and units)	Source	Degrees of freedom	Sum of squares	%SS [a]	*p*-value
County-level maize yield ($Mg\ ha^{-1}$)	TED	19	4201	81%	<0.01
	Year	9	984	19%	<0.01
	TED x year	167	14	<1%	<0.01
	Residual	1699			

[a] %SS: proportion of sum of squares relative to the non-error total sum of squares.

This finding was consistent with the analysis of the field-level soybean yield and management data (tillage, maturity groups, and foliar pesticide) performed over 17 TEDs. The analysis of field-level soybean data indicated that ≥80% of the treatment sum of squares was attributable to TEDs, in all cases explaining more than four times more than the contribution of year and TED x year (Table S3). Altogether, the findings from the analysis performed for two crops (maize and soybean) at two different spatial scales (county and field) indicate that the TEDs framework can be used to reliably delineate climate-soil domains that account for variation in yield and management practices in a majority of the years.

Table S3 – Analysis of variance of the TED framework across years using field-level soybean yield and management data. *Analysis of variance for seed yield, tillage, variety maturity group, and in-season foliar pesticide (fungicide and/or insecticide) reported for producer soybean fields sown with soybean during 2014–2016 years across 17 technology extrapolation domains (TEDs) located in the US Corn Belt region. Seed yield and management practices were collected from 3276 producer fields through surveys.*

Variable (and units)	Source	Degrees of freedom	Sum of squares	%SS [a]	*p*-value
Seed yield	TED	16	134692	81%	<0.01
(Mg ha^{-1})	Year	2	17941	10%	<0.01
	TED x year	32	14600	9%	<0.01
	Residual	3275			
Tillage	TED	16	66	80%	<0.01
(% tilled fields)	Year	2	1	1%	0.30
	TED x year	32	16	19%	<0.01
	Residual	3193			
Maturity group	TED	16	864	99%	<0.01
(unitless)	Year	2	1	<1%	0.08
	TED x year	32	9	1%	0.03
	Residual	3001			
Foliar pesticide	TED	16	76	81%	<0.01
(% treated fields)	Year	2	1	1%	0.07
	TED x year	32	17	18%	<0.01
	Residual	3275			

[a] %SS: proportion of sum of squares relative to the non-error total sum of squares.

S5. Technology extrapolation domains application

Tool to guide evaluation and scaling out of agronomic technologies

The NutrientStar® program seeks to assess performance of fertilizer products to improve nutrient use efficiency and reduce negative environmental impact from nutrients applied in crop production (www.nutrientstar.org). In 2015, NutrientStar® established a set of 96 field experiments in the north-central US region. The location of these trials (Supplementary Figure S4) was used as a case study here for evaluating the potential of using the TED framework to more efficiently select trial sites to achieve greater coverage of maize area in unique TED zones. The 96 sites were located within 19 moderate resolution TEDs because there was more than one site per TED in many cases (Figure 4B). Potential use of the TED scheme to improve current experimental site selection was assessed by comparing the actual trial network against two hypothetical designs: one aimed to reach same coverage with the smallest number of sites (Figure 4C), and another aimed to maximize cropland coverage with the same number of sites (Figure 4D). Maize area within the actual and the two hypothetical trial locations were computed as the sum of maize area across all TEDs where, at least, one experimental site was located.

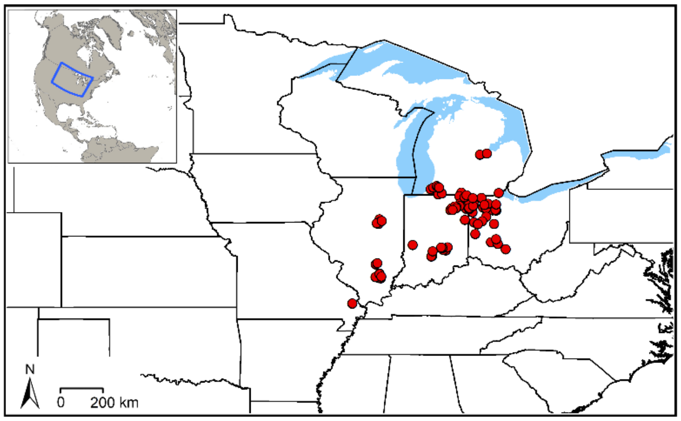

Figure S4. Field trial location. *Field trial network designed to test a fertilizer-related product which was used here as a case study for evaluating potential of using the technology extrapolation domain framework to more efficiently select trial location. Red dots indicate location of the 96 sites.*

Cross-country technology transfer

An analysis based on the GYGA climate zone scheme conducted for rainfed wheat in Australia and Argentina revealed that these two countries shared a relevant climate zone for wheat production (Aramburu Merlos et al., 2015; Gobbett et al., 2017), which is mostly located in the north-eastern grain zone of Australia and in central Argentina (Figure 5). Unsurprisingly, a comparison of annual variation of maximum and minimum temperature, solar radiation, rain, and potential evapotranspiration[2] (Allen et al., 1998) between two representative locations of mentioned climate zone (Gunnedah, Australia and General Pico, Argentina) showed a similar pattern of weather variation throughout the year (Figure S5). Likewise, PAWHC was 252 mm for Gunnedah and 216 mm for General Pico; this difference is smaller than the 50 mm range used to build the moderate resolution TED. In other words, the two sites are located within the same TED. Although climate and soils are very similar, cropping system is different between countries. While the Argentinean system includes 1.5 crops per year (2-yr soybean-wheat-soybean), the Australian system has, on average, less than one crop per year (predominantly wheat and other cereal crops) (Andrade and Satorre, 2015; Hochman et al., 2014; Martin et al., 1988). Due to its higher crop intensity, the Argentine TED has higher crop yield, relative to the Australian system, when yields are expressed on an annual basis.

[2] Grass-referenced potential evapotranspiration was estimated at both locations using Penman-Monteith-FAO56, while relative humidity, used for ETo estimation, was derived from daily Tmax and Tmin.

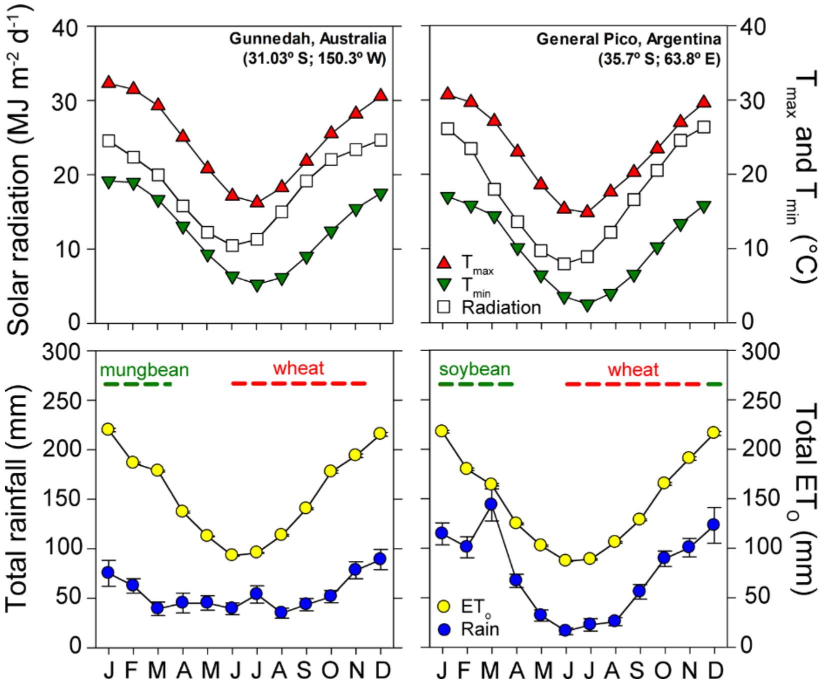

Figure S5. Annual weather variation for the same technology extrapolation domain in Australia and Argentina. *Data points are 29-y (1983-2011) monthly averages of maximum (Tmax) and minimum (Tmin) temperature, solar radiation, grass-referenced evapotranspiration (ET_0) and total rainfall. ET_0 was estimated at both locations using Penman-Monteith-FAO56 method (Allen et al., 1998). Dashed lines roughly indicate the extention of the crop seasons. Analysis was based on measured daily weather data collected in meteorological stations located*

We evaluated the feasibility of increasing annual productivity and resource capture of the Australian cropping system by increasing its crop intensity by inclusion of a summer legume (mungbean) in the traditional wheat-fallow system. Average water-limited yield potential and CV of wheat and mungbean, within each of the two rotations (wheat-fallow and wheat-mungbean), were simulated using the Agricultural Production Systems Simulator (APSIM; McCown et al., 1996) based on long-term (1983-2012) measured daily weather data and the dominant soil type within the target TED. APSIM is a modular modelling framework developed to simulate biophysical process in farming systems, in particular where there is interest in the economic and ecological outcomes of management practices in the face of climatic risk. It

contains a suite of modules that include a diverse range of crops, pastures and trees, soil processes including water balance, N and P transformations, soil pH, erosion and a full range of management controls. APSIM has been used in a broad range of applications, including support for on-farm decision making, farming systems design for production or resource management objectives, seasonal climate forecasting, risk assessment for government policy making and as a guide to research and education activities (Keating et al., 2003 and references therein).

The wheat-mungbean rotation was analysed with or without a threshold value (60 mm) of stored soil water at sowing to trigger mungbean sowing. This threshold value was set to reduce the risk associated with low available soil water at sowing on crop yields and its variation in a semiarid environment (Nielsen et al., 2009). The three crop rotation options (wheat-fallow, wheat-mungbean in all years, and wheat-mungbean rotation where mungbean is an opportunistic crop depending on soil water status) were compared on the basis of average annual income from grain purchase (USD ha^{-1} yr^{-1}), its CV, and water and solar radiation availability and capture (Table S4). Net income was estimated based on average annual gross income minus variable costs and overhead costs. Cost prices assumption were (i) wheat price: 205 USD Mg^{-1}; variable costs: 340 USD ha^{-1}; and (ii) mungbean price: 530 USD Mg^{-1}; variable cost: 376 USD ha^{-1}. Overhead costs were considered 152 USD ha^{-1}, regardless of crop rotation and crop intensity. Costs and prices were based on New South Wales (Australia) Government reports (www.dpi.nsw.gov.au), assuming 5-year (2011-2015) average prices.

Table S4 – Water and solar radiation availability and capture for the three cropping systems in Australia. *Average (± standard deviation) annual precipitation and incident solar radiation calculated based on historical measured weather data (2005-2014) collected at Gunnedah (Australia). Proportion of annual total water and solar radiation captured by crops (expressed in percentage) was calculated for each cropping systems.*

Cropping system	Precipitation (mm y^{-1})	Incident solar radiation (MJ m^{-2} y^{-1})	Water capture (%)	Solar radiation capture (%)
Continuous wheat	644 (161)	6749 (235)	27	16
Wheat-mungbean			43	24
Wheat-mungbean (60-mm threshold)			42	23

References

Allen, R. G., et al., Crop evapotranspiration: guidelines for computing crop water requirements. FAO Irrigation and Drainage Paper. No. 56. FAO, Rome, Italy, 1998.

Andrade, J. F., Satorre, E. H., 2015. Single and double crop systems in the Argentine Pampas: Environmental determinants of annual grain yield. Field Crops Research. 177, 137-147.

Aramburu Merlos, F., et al., 2015. Potential for crop production increase in Argentina through closure of existing yield gaps. Field Crops Research. 184, 145-154.

Danvi, A., et al., 2016. A spatially explicit approach to assess the suitability for rice cultivation in an inland valley in central Benin. Agricultural Water Management. 177, 95-106.

FAO, Report on the agro-ecological zones project. World Soil Resources Report 48, Rome, Italy, 1978.

Fischer, G., et al., Global agro-ecological assessment for agriculture in the 21st century: Methodology and Results. IIASA, Laxenburg, Austria, 2002.

Gobbett, D. L., et al., 2017. Yield gap analysis of rainfed wheat demonstrates local to global relevance. Journal of Agricultural Science. 155, 282-299.

Hijmans, R. J., et al., 2005. Very high resolution interpolated climate surfaces for global land areas. International Journal of Climatology. 25, 1965-1978.

Hochman, Z., et al., 2014. Crop sequences in Australia's northern grain zone are less agronomically efficient than implied by the sum of their parts. Agricultural Systems. 129, 124-132.

Keating, B. A., et al., 2003. An overview of APSIM, a model designed for farming systems simulation. European Journal of Agronomy. 18, 267-288.

Kleinbaum, D. G., et al., 1998. Applied regression analysis and other multivariate methods. Duxbury Press.

Kouadio, L., Newlands, N. K., 2015. Building capacity for assessing spatial-based sustainability metrics in agriculture. Decision Analytics. 2, 2.

Licker, R., et al., 2010. Mind the gap: how do climate and agricultural management explain the 'yield gap' of croplands around the world? Global Ecology and Biogeography. 19, 769-782.

Martin, R., et al., 1988. Survey of farm management practices of the northern wheat belt of New South Wales. Australian Journal of Experimental Agriculture. 28, 499-509.

McCown, R. L., et al., 1996. APSIM: a novel software system for model development, model testing and simulation in agricultural systems research. Agricultural Systems. 50, 255-271.

Nielsen, D. C., et al., 2009. The variable response of dryland corn yield to soil water content at planting. Agricultural Water Management. 96, 330-336.

Padbury, G., et al., 2002. Agroecosystems and land resources of the Northern Great Plains. Agronomy Journal. 94, 251-261.

Singh, V. P., et al., Ecosystem analysis-based methodology for technology extrapolation. In: V. Balasubramanian, et al., Eds.), Resource Management in Rice Systems: Nutrients. Springer Netherlands, Dordrecht, 1999, pp. 213-229.

Soil Survey Staff, National Value Added Look Up (valu) table database for the Gridded Soil Survey Geographic (gSSURGO) Database for the United States of America and the Territories, Commonwealths, and Island Nations served by the USDA-NRCS. United States Department of Agriculture, Natural Resources Conservation Service, 2016.

USDA-FSA-APFO, The Geospatial Data Gateway. National Elevation Dataset 10 Meter. www.datagateway.nrcs.usda.gov. 2016.

USDA-NASS, USDA-National Agricultural Statistics Service (NASS), Crop U.S. State and County Databases.www.nass.usda.gov. 2016.

USDA-NASS, USDA-National Agricultural Statistics Service (NASS), National Cultivated Layer. www.nass.usda.gov. 2017.

van Bussel, L. G. J., et al., 2015. From field to atlas: Upscaling of location-specific yield gap estimates. Field Crops Research. 177, 98-108.

van Wart, J., et al., 2013. Use of agro-climatic zones to upscale simulated crop yield potential. Field Crops Research. 143, 44-55.

Wood, S., Pardey, P. G., 1998. Agroecological aspects of evaluating agricultural R&D. Agricultural Systems. 57, 13-41.

Zomer, R. J., et al., 2008. Climate change mitigation: a spatial analysis of global land suitability for clean development mechanism afforestation and reforestation. Agriculture Ecosystems & Environment. 126, 67-80.
